# The influence of mindfulness meditation combined with progressive muscle relaxation training on the clinical efficacy and quality of life of patients with sarcopenia receiving haemodialysis: a randomised controlled trial

**DOI:** 10.1186/s12906-024-04485-3

**Published:** 2024-05-17

**Authors:** Yong-Yao Wu, Yi-Yi Gao, Jing-Qiao Wang, Chao Zhang, Peng-Jie Xu, Jiang Liu, Ri-Zhen Yu, Hao-Jie Zhang

**Affiliations:** 1 Department of Nephrology, Ningbo Medical Center Lihuili Hospital, No. 1111 Jiangnan Road, Yinzhou District, Ningbo, 315099 Zhejiang China; 2 Urology & Nephrology Center, Department of Nephrology, Zhejiang Provincial People’s Hospital (Affiliated People’s Hospital), Hangzhou Medical College, Hangzhou, 310014 Zhejiang China

**Keywords:** Mindfulness meditation, Progressive muscle relaxation, Uremic sarcopenia, Maintenance haemodialysis, Quality of life

## Abstract

**Objective:**

To study the effect of mindfulness meditation combined with progressive muscle relaxation training on the clinical efficacy and quality of life in patients with sarcopenia receiving maintenance haemodialysis (MHD).

**Methods:**

Eligible patients with sarcopenia in our hospital were randomly assigned to a control group (*n* = 24) and an intervention group (*n* = 25). The control group received conventional dialysis treatment, while the intervention group underwent mindfulness meditation combined with progressive muscle relaxation training during the interdialysis period in addition to conventional dialysis treatment. The effect of the intervention was evaluated after 12 weeks.

**Results:**

There were no significant differences in the baseline values of various parameters between the two groups. Exercise capacity (sit-to-stand test,handgrip,time to 10 sit-ups) significantly improved in the intervention group after 12 weeks (32.68 ± 8.32 vs 26.50 ± 6.83; 37.42 ± 10.12 vs 28.12 ± 8.51; 19.8 ± 5.40 vs 25.29 ± 7.18) (*p* < 0.05). In terms of the kidney disease quality of life (KDQOL^TM^) score, all other dimensions except sexual function, social functioning, burden of kidney disease and work status dimensions showed significant improvement compared to the baseline (*p* < 0.05). In the control group, only the dialysis staff encouragement (DSE) and patient satisfaction (PS) dimensions showed slight improvements compared to the baseline (*p* > 0.05). When compared with the control group, the intervention group showed significant improvements in 10 dimensions of exercise capacity and KDQOL^TM^ scores for physical function, role-physical, general health, energy, symptom/problem list, sleep, DSE, pain, cognitive function, emotional well-being and patient PS after 12 weeks (61.30 ± 5.38 vs 42.98 ± 5.73; 57.50 ± 3.55 vs 50.70 ± 3.62) (*p* < 0.05). Some inflammatory markers, such as the levels of interleukin-6 and high-sensitivity C-reactive protein (30.29 ± 2.96 vs 17.65 ± 3.22; 8.93 ± 0.99 vs 3.02 ± 0.34), showed a decrease during the intervention, while albumin and prealbumin levels were significantly increased compared with the baseline (30.62 ± 1.65 vs 35.60 ± 1.68; 0.32 ± 0.05 vs 0.44 ± 0.07) (*p* < 0.05).

**Conclusion:**

Combined intervention training can improve the motor ability and quality of life of patients with sarcopenia within a short period of time.

**Supplementary Information:**

The online version contains supplementary material available at 10.1186/s12906-024-04485-3.

## Background

Sarcopenia is a syndrome characterised by a progressive decline in muscle mass, strength and physical function and is expected to affect more than 200 million patients worldwide by 2050 [1]. As of 2017, there have been 132 million CKD patients in China [2]. Due to dietary restrictions and hypercatabolism, the decline of muscle mass, function and strength occurs earlier and progresses faster and is referred to as ‘uremic sarcopenia’ [3]. The clinical manifestations often included a slowed walking speed and even difficulty completing daily activities such as sitting up and turning [3]. The risk of sarcopenia in patients with uraemia is gradually increasing, with an incidence of 63% in the elderly haemodialysis population and a significant increase in cardiovascular, hospital and even death events [3, 4]. A survey of sarcopenia in patients receiving maintenance haemodialysis (MHD) by the Second Military Medical University in Nanjing in 2014 [5] showed that the overall incidence of sarcopenia was 13.7% in 131 patients receiving MHD, 33.3% in patients over 60 years of age and 88.9% in patients with sarcopenia at 1 year. Its pathogenesis is related not only to genetic, environmental, ageing and lack of exercise factors but also to inflammation, malnutrition and mood disorders [6–8]. The condition is caused by the interaction of multiple risk factors and related pathogenic mechanisms.

There are many studies on sarcopenia abroad. The harm it can cause to patients receiving MHD has been recognised, and the interventions for sarcopenia in patients receiving MHD have been studied. Research on sarcopenia in China is still in its infancy. The current treatment of patients with sarcopenia receiving MHD is mainly to provide nutritional therapy, as well as exercise and drug therapy to improve their exercise capacity and quality of life(QOL) . Current studies have rarely focused on emotional problems in patients with sarcopenia receiving MHD and related interventions have been poorly investigated. Symptoms of depression and anxiety affect approximately 50% of patients receiving MHD [9–11] and are associated with a reduced quality of life [12–15], increased haemodialysis noncompliance [16], suicidal behaviour [17], medical comorbidities and mortality [12–15].

Mindfulness meditation training is a cognitive method that teaches patients to use or stimulate their own intrinsic strength through a body perception approach that enhances the patient's self-regulation ability and emotional cognitive therapy [18]. Mindfulness meditation training has been applied in many types of psychiatric conditions [19–21] with good results. For patients receiving haemodialysis in particular, a study demonstrated that Benson relaxation (a Transcendental Meditation technique) delivered by a caregiver had a modest positive benefit for anxiety [22]. A limitation of this study was, however, that patients with major medical comorbidities, physical disabilities and psychiatric histories were excluded, rendering the results difficult to generalise to the typical haemodialysis population. The results of Wang Bingjing's [23] study showed that short-term mindfulness meditation training reduced anxiety and improved sleep quality and coping style in patients with GAD. In addition, the positive findings of various studies demonstrated that mindfulness is a promising method to use for patients undergoing haemodialysis as a way to manage stress and improve their quality of life [24–26]. Therefore, a methodologically sound, scalable and generalisable clinical trial of mindfulness meditation for depression and anxiety symptoms in patients on haemodialysis should be conducted [27, 28].

Progressive muscle relaxation training is a method that employs ongoing relaxation training [29]. This type of training guides patients to effectively observe changes occurring in their muscles from a state of tension to relaxation, achieved by training the muscles in various parts of the body, thereby indirectly improving their psychological state and adjusting disturbances in the functioning of the patient's body [30]. Progressive muscle relaxation training is currently applied to diseases such as cancer, fibromyalgia and insomnia to curb vomiting and improve aspects related to quality of life, pain and insomnia in patients [31–33].

At present, there are few clinical reports on the combined use of these two techniques in the treatment of uremia-associated sarcopenia. This study aimed to observe the effect of mindfulness meditation combined with progressive muscle relaxation training on the clinical efficacy and quality of life in patients with uraemic sarcopenia and to preliminarily explore the underlying mechanism, whether mindfulness meditation combined with progressive muscle relaxation training intervention achieves the therapeutic effect by reducing inflammatory parameters.

## Objectives and methods

### Study population

In this study, we first investigated the occurrence of sarcopenia in patients receiving MHD using the following sample size calculation formula: *n = μ*^*2*^_*α/2*_*π (1 − π) / δ*^*2*^, where the *δ* value was 5%, *μ*_*α/2*_ = 1.96, *π* was set to 33.3% [34] and the calculated sample size to be investigated was 341. According to the diagnostic indicators of sarcopenia, 49 patients who were finally diagnosed with sarcopenia were included in the study.

### Inclusion and exclusion criteria

Inclusion criteria: Patients who met the sarcopenia criteria established by the Asian Working Group for Sarcopenia [35] in 2014; stable condition and generally good health status; good compliance and the ability to collaborate well with physicians; undergoing haemodialysis three times per week for at least three months with a KT/V ≥ 1.2; no significant cardiovascular or cerebrovascular complications (such as severe heart failure, severe arrhythmias, angina and cerebrovascular diseases). Patients with sarcopenia who met the clinical diagnostic criteria were selected to ensure the quality of the included patients; patients with stable disease and no significant cardiovascular and cerebrovascular complications were also selected to avoid changes in their condition and their disease affecting the test results; the inclusion criteria required haemodialysis three times a week for at least 3 months and KT/V ≥1.2 for the selection of patients with sarcopenia that occurred during dialysis.

Exclusion criteria: Inability to complete a handgrip strength (HGS) test, a 6-meter walk test, 10 sit-to-stand test repetitions and where using bioelectrical impedance analysis was not possible due to contraindications; co-existence of mental illness or severe cognitive or communication disorders. Because exercise capacity tests and scales had to be completed, patients who could not complete these and could not communicate normally were excluded to avoid affecting the test results.

Elimination criteria: Participants who did not follow the study protocols and who failed to complete it; participants for whom the assessment of improvement effects was not possible or whose data were incomplete, thereby affecting the assessment of efficacy.

This study was approved by the hospital ethics committee and informed consent was obtained from all participants. The study's purpose was explained and voluntary participation was ensured.

### Grouping methods

This study was a randomised controlled trial. Patients who met the inclusion criteria were randomly assigned to the intervention (*n* = 24) or the control (*n* = 25) group according to a random number table. Except for the researchers who completed the grouping, the remaining researchers and study participants were not made aware of the grouping information before assigning the intervention. Two patients in the intervention group and two patients in the control group dropped out during the study (automatic withdrawal for reasons given as ‘other’). Finally, 45 patients completed the study with a completion rate of 91.8%. Dropouts were 8.0% and 8.3% in the intervention and control groups, respectively. A total of 49 questionnaires were distributed and all 49 were collected, yielding a response rate of 100% (CONSORT 2010 checklist; see Attachment 1).

Both groups underwent comprehensive assessments and, based on the assessment results, received health education and dietary guidance including the following recommendations: aim for a daily total calorie intake of 30–35 kcal/kg; consume 1.0–1.2 g/kg of protein per day, with restrictions on potassium, phosphorus and salt intake; maintain weight gain during dialysis within the range of 3%–5% of dry weight; receive psychological support and manage negative emotions based on psychological assessments; encourage patients to engage in appropriate daily exercise, following the principle of gradual progression, unless there are contraindications to exercise; maintain haemoglobin levels within the range of 100–120 g/L; control blood pressure around 140/90 mmHg.

#### Control group

Patients in the control group received conventional treatment, mainly dietary guidance, oral food protein, fatty acids, vitamin D and antioxidant nutrients to strengthen nutrition to maintain a balanced diet and adequate nutrition in daily life.

#### Intervention group

In addition to conventional treatment, mindfulness meditation combined with progressive muscle relaxation training was implemented during the interdialytic period. A multidisciplinary intervention team was established, consisting of two meditation instructors, three physicians and two specialised nurses, all of whom possessed good communication skills, coordination abilities and emergency response capabilities. All team members received a 2-month training course on the correct movements and processes (operational training) for progressive muscle relaxation training and mindfulness meditation training, as well as an introduction to relevant theoretical knowledge (theoretical training). Training was conducted 3 times a week for 2 months, 1 day per session, with progressive muscle relaxation training in the morning and mindfulness meditation training in the afternoon. Their training effect was assessed at the end of the training and only patients who passed the assessment were eligible to participate in the study.

Joint intervention measures: This included two parts, the first of which was progressive muscle relaxation training. The second part was mindfulness meditation training. Under the guidance of a meditation instructor, daily online video lessons were conducted, introducing patients to the theoretical knowledge and specific methods of mindfulness meditation and muscle relaxation training. After 4 weeks, patients were expected to continue practising at home, conducting self-practice twice a day for 15–20 minutes each time during the haemodialysis period for a total of 12 weeks.

The standardised mindfulness meditation manual is as follows [36].

Preparing for training: Find a comfortable and quiet position to sit down and engage in slow breathing through your nose and mouth.

Training method [36]: Under guidance, perform muscle contraction and relaxation exercises, including continuous relaxation of the 10 parts of the body from head to toe.

① Head relaxation: Inhale and forcefully furrow your brow, hold for 5 seconds, exhale and relax. Inhale and tightly close your eyes, hold for 5 seconds, then exhale and relax. Inhale and scrunch up your nose and cheek muscles, hold for 5 seconds, then exhale and relax. Inhale and press your tongue against your upper front teeth, open your mouth as wide as possible, tilt your head back, hold for 5 seconds, and then exhale and relax. ② Neck muscle relaxation: Inhale and gently tilt your head backwards, as if looking up at the ceiling; hold for 5 seconds, exhale and relax. Pay attention to the sensation of muscle relaxation. ③ Shoulder muscle relaxation: Place your arms by your sides, inhale, and try to lift your shoulders upward as much as possible, hold for 5 seconds, then exhale and relax. ④ Arm muscle relaxation: Place your palms facing upward on the armrests of a chair, inhale, and make a fist, keeping your hands and forearms tense for 5 seconds, then exhale, and relax. Then, raise both arms to the sides and open them wide as if stretching your chest, feel the tension in your arms for 5 seconds, and then exhale and relax. ⑤ Chest muscle relaxation: Bring your shoulders forward, inhale, and tense the muscles around your chest; hold for 5 seconds, then exhale and relax. ⑥ Back muscle relaxation: Inhale and forcefully expand your shoulders backwards; feel the tension in your back muscles for 5 seconds, then exhale and relax. Arch your back backwards, making an effort to lift your chest; squeeze the back muscles for 5 seconds, then exhale and relax. ⑦ Abdominal muscle relaxation: Inhale and tighten your abdominal muscles as if someone is about to punch you in the stomach; hold the contraction for 5 seconds, then exhale and relax.⑧ Hip muscle relaxation: Inhale and clench your buttock muscles, tighten your anal sphincter, and hold the tension for 5 seconds, then exhale and relax. ⑨ Leg muscle relaxation: Inhale and tense your legs, lift them straight up about 20 centimetres off the ground, hold for 5 seconds, then exhale and relax. ⑩ Toe muscle relaxation: Inhale and slowly curl your toes downward as if gripping the ground; hold for 5 seconds, then exhale and relax. Slowly raise your toes upward, maintain the tension for 5 seconds, then exhale and relax.

The term ‘relaxation’ refers to actively experiencing the comfortable and relaxed sensation that occurs after the muscles have released tension, such as a feeling of warmth, mild soreness or softness.

After muscle relaxation training, specific interventions were implemented according to the standardised mindfulness meditation manual as follows [37].

① Sitting posture: The individual chose a seated position that was both relaxed and stable. They crossed their knees or sat with the soles of their feet facing upward. They closed their eyes and ensured that their spine was straight, maintaining a relaxed posture without being too rigid. ② Body scan: While breathing, the individual shifted their attention to different parts of their body, noting any areas that felt slightly tense or uncomfortable, observed them and accepted the sensations. Then they slowly shifted their attention away from those areas. ③ Mindful breathing: The individual focused their attention on their breath, taking deep breaths into their abdomen and exhaling slowly. They noticed the sensations of inhalation and exhalation. ④ Mindfulness meditation: In a quiet environment, with a relaxed body, the individual focused their attention on the thoughts, mental images and emotions that arose in their mind. They observed these thoughts, images and emotions without judgment, cultivating objective awareness. They recognised negative emotions and responded to them appropriately. They also objectively recognised thoughts and events that arose in their mind while feeling the rhythm of their breathing and the relaxation of their body. ⑤ Bringing the attention back to the breath: The individual focused their attention on their breath, from head-to-toe, allowing each breath to become calmer and deeper. They experienced a sense of natural relaxation and slowly opened their eyes as they exhaled.

After the training, any issues encountered during each exercise session were recorded and documented for review and discussion in the following session. This process aimed to help patients integrate mindfulness into their daily lives, experience self-acceptance and the acceptance of others and achieve harmonisation with the surrounding environment.

### Observation indicators and methods

The indicators of both groups were observed before and after the 12-week intervention. This included assessments of physical fitness, Kidney Disease-Related Quality of Life Short Form (KDQOL-SF^TM^) scores, laboratory tests and complications. The differences in indicator changes between the two groups were compared. Prior to the experiment, the purpose and methods were explained to the participants, and they were familiarised with the testing procedures and environment and encouraged to complete the exercise testing as thoroughly as possible. Speed adjustments (slowing down or taking short breaks) were made if necessary.

#### Physical fitness

The HGS, usual gait speed (UGS) and time taken to complete 10 sit-to-stand repetitions were tested [38].

① Handgrip strength: The grip strength was measured three times using the CAMRY Hand Grip Dynamometer (CAMRY, EH101, Shenzhen Linghao Sports Products Co., Ltd.) and the average value was calculated.
② Usual gait speed: The time taken for both groups of patients to walk in a straight line for 6 meters at a normal walking speed was recorded. Three measurements were recorded during the testing process, with a 2-minute rest interval between each measurement. The average value was then calculated.
③ Time taken for 10 sit-to-stand repetitions: The time taken for participants to repeatedly stand up and sit down from a chair of standard height (10 repetitions) was recorded.

Vital signs such as heart rate, blood pressure and respiratory rate were recorded before and after the experiment. If participants experienced significant symptoms such as dizziness, angina or shortness of breath, the tests were immediately stopped.

#### Kidney disease-related quality of life short form scores

The KDQOL-SF^TM^ [39] includes two parts: kidney disease and dialysis-related quality of life (KDTA) (KDQOL-SFTM1.3) and general health (GH)-related quality of life (SF-36). The SF-36 questionnaire evaluates the subjective health-related quality of life across eight dimensions, including physical function (PF), role-physical (RP), bodily pain (Pain), GH, energy/fatigue (Energy), social functioning (SoF), role limitations-emotional (RE) and emotional well-being (EWB). The KDTA includes 43 items specific to kidney disease, divided into 11 dimensions as follows: symptom/problem list (SPL), effects of kidney disease (EKD), burden of kidney disease (BKD), work status (WS), cognitive function (CF), quality of social interaction (QSI), social support, sexual function (SexF), sleep (SL), patient satisfaction (PS) and dialysis staff encouragement (DSE).

The scores for each domain indicate that the higher the score, the better the quality of life in that particular area. The scores ranged from 0 to 100. Under the guidance of the researchers, the patients are asked to complete the questionnaire within 30 minutes before the combined intervention and again after 12 weeks.

#### Laboratory tests

Fasting venous blood samples were collected to measure the levels of interleukin-6 (IL-6), high-sensitivity C-reactive protein (hs-CRP), tumor necrosis factor (TNF), albumin (ALB) and prealbumin (PA) before and after the intervention.

Using a body composition analyser (InBody 770, Biospace), the skeletal muscle index (SMI) and skeletal muscle mass (SMM) were measured.

During the study period, any complications related to the combined intervention are recorded.

### Statistical methods

The SPSS 22.0 statistical software was used in this study. The measurement data that met a normal distribution is expressed by (x̄ ± s). The between-group comparisons employed an independent sample t-test, and within-group comparisons used a paired t-test. Data that did not meet a normal distribution is expressed by M (IQR). Between-group comparisons employed a Mann–Whitney U test, and within-group comparisons used a Wilcoxon symbolic rank test. The count data are expressed in n (%), and a chi-squared test was used for comparison between groups. According to the test level, *p* < 0.05 indicated a statistically significant difference.

## Results

### Baseline characteristics of patients

The patients received haemodialysis three 3 per week, and their average age was 58.8 ± 14.9 years. The average duration of haemodialysis was 2.8 ± 0.7 years. There were no significant differences in baseline characteristics between the two groups in terms of various indicators (*p* > 0.05) (Table 1).

**Table 1:** Characterization of study population (*n* =49)

	Te-Group (*n*=25)	Con-Group (*n*=24)	X^2^/t/z	P
Sex(%)			0.168	0.682
Men	19(76.0)	17(70.8)		
Women	6(24.0)	7(29.2)		
Age(year)	45.52±4.43	45.46±5.12	0.045	0.964
MHD duration	59.16±18.85	54.46±15.98	0.940	0.352
Educational level(%)			1.380	0.240
Moderate and high	16(64.0)	19(79.2)		
Low	9(36.0)	5(20.8)		
Employment status(%)			0.698	0.404
Employed	9(36.0)	6(25.0)		
Unemployed	16(64.0)	18(75.0)		
Marital status(%)			0.525	0.469
Married	13(52.0)	10(41.7)		
Not married	12(48.0)	14(58.3)		
Income status(%)			1.041	0.308
High, moderate and above	12(48.0)	15(62.5)		
Low	13(52.0)	9(37.5)		
Medical insurance(%)			0.163	0.686
Good or moderate	17(68.0)	15(62.5)		
Poor	8(32.0)	9(37.5)		
Systolic pressure(mmHg)	151.72±7.25	150.42±7.16	0.633	0.530
Diastolic pressure(mmHg)	89.80±10.41	86.88±15.03	0.795	0.431
Dry weight(Kg)	58.9(57.5-66.8)	60.6(57.0-62.9)	-0.730	0.465
Etiology of ESRD(%)			7.106	0.418
purpura nephritis	3(12.0)	1(4.2)		
Gouty nephropathy	1(4.0)	2(8.3)		
Diabetes	2(8.0)	3(12.5)		
LN	2(8.0)	1(4.2)		
Obstructive nephropathy	0(0.0)	3(12.5)		
Membranous nephropathy	2(8.0)	1(4.2)		
Hypertension	1(4.0)	2(8.3)		
Other	14(56.0)	11(45.8)		

Explanation: From the above table, it can be seen that after comparison between groups, there was no statistically significant difference in general data between the two groups, indicating comparability between the groups. MHD: Maintenance haemodialysis, ESRD: end stage renal disease

### Changes in various indicators of physical abilities

After 12 weeks, the intervention group showed significant improvements in all physical ability indicators compared to before the intervention (32.68 ± 8.32 vs 26.32 ± 6.16; 37.42 ± 10.12 vs 27.36 ± 8.10; 19.8 ± 5.40 vs 24.60 ± 6.49), and the differences were statistically significant (*p* < 0.05). In contrast, the control group showed no significant improvements. When comparing the intervention group with the control group, all physical ability indicators in the intervention group showed significant improvements (32.68 ± 8.32 vs 26.50 ± 6.83; 37.42 ± 10.12 vs 28.12 ± 8.51; 19.8 ± 5.40 vs 25.29 ± 7.18), and the differences were statistically significant (*p* < 0.05) (see Table 2). No trial-related complications occurred between the two groups.

**Table 2:** Primary monitoring indicators (exercise capacity,SMM and SMI)

	Te-Group		Con-Group	
	Baseline	End of study	Baseline	End of study
sit-to-stand test (S)	26.32±6.16	32.68±8.32^△☆^	26.83±8.25	26.50±6.83
grip strength (Kg)	27.36±8.10	37.42±10.12^△☆^	27.29±8.44	28.12±8.51
Time to10 sit-ups (s)	24.60±6.49	19.8±5.40^△☆^	24.79±6.97	25.29±7.18
SMM(kg)	22.20±2.86	23.58±2.41	22.90±2.51	23.18±2.48
SMI(kg/m^2^)	7.08±1.23	7.43±1.47	6.96±0.95	7.13±1.69

Data are mean ± SD. Within-group comparison with the baseline ^△^*P*<0.05; Compared with control group after exercise therapy. ^☆^*P*<0.05. *SMI* Skeletal muscle index, *SMM* skeletal muscle mass

Explanation: From the above table, it can be seen that after inter group comparison, there was no statistically significant difference between the two groups before the intervention. The intervention group dared to have statistically significant differences in sit to stand test, grip strength, and Time to 10 sit ups before and after the intervention, while the control group had no statistically significant differences before and after the intervention. After the intervention, there were statistically significant differences in sit to stand test, grip strength, and Time to 10 sit ups between the groups. SMM There was no statistically significant difference in SMI

### Changes in inflammation and nutrition indicators

After 12 weeks, in the intervention group, some inflammation indicators (IL-6, hs-CRP) decreased (30.29 ± 2.96 vs 17.65 ± 3.22; 8.93 ± 0.99 vs 3.02 ± 0.34), while nutrition indicators (ALB, PA) increased significantly compared to before the intervention (30.62 ± 1.65 vs 35.60 ± 1.68; 0.32 ± 0.05 vs 0.44 ± 0.07). The differences were statistically significant (*p* < 0.05) (see Table 3).

**Table 3:** Primary monitoring indicators (KDQOL-SF^TM^)

	Te-Group		Con-Group	
	Baseline	End of study	Baseline	End of study
SF-36	40.91±4.65	61.30±5.38^△☆^	41.08±5.65	42.98±5.73
PF	58.00±10.21	84.00±5.59^△☆^	58.96±9.55	60.42±8.96
RP	26.00±19.74	54.00±20.00^△☆^	26.04±18.77	27.08±20.74
Pain	57.50±16.46	84.80±9.54^△☆^	56.98±14.50	59.48±15.60
GH	33.80±11.02	49.00±12.33^△☆^	34.37±10.77	35.21±10.58
Energy	41.00±11.90	54.40±9.50^△☆^	40.42±12.15	41.67±12.04
SocF	35.50±14.74	37.00±12.23	35.94±14.42	35.94±15.77
RE	22.67±20.91	40.00±25.46^△^	23.61±20.80	30.56±21.80
EWB	52.80±8.33	87.20±6.22^△☆^	52.33±8.66	53.50±7.81
KDTA	46.97±2.93	57.50±3.55^△☆^	47.12±2.83	50.70±3.62
SPL	43.50±5.32	62.25±8.62^△☆^	42.71±4.47	44.10±5.41
EKD	37.63±5.45	41.75±6.74^△^	38.15±4.51	40.49±5.49
BKD	27.25±10.03	28.25±8.68	27.34±9.72	27.86±8.64
WS	26.00±29.30	28.00±29.15	27.08±29.41	27.08±32.9
CF	65.33±8.39	72.00±7.70^△^	65.83±8.41	68.89±8.26
QSI	61.60±9.87	67.47±8.01^△^	62.78±8.32	64.72±9.92
SoS	71.33±17.69	81.33±13.02^△^	71.52±17.36	75.00±14.75
SexF	15.00±8.84	17.00±9.46	15.10±8.23	16.67±10.21
sleeps	34.90±7.38	55.40±9.75^△☆^	34.48±7.70	35.73±7.05
DSE	71.50±17.87	91.00±9.21^△☆^	70.83±17.55	80.73±13.78^△^
PS	62.67±18.18	88.00±14.85^△☆^	62.50±18.55	76.39±16.24^△^

Data are mean ± SD. Within-group comparison with the baseline ^△^*P*<0.05; Compared with control group after exercise ^☆^*P*<0.05. *PF* Physical function, *RP* Role-physical, *GH* General health, *RE* Role limitations-emotional, *EWB* Emotional well-being, *SPL* symptom/problem list, *EKD* Effects of kidney disease, *BKD* Burden of kidney disease, *WS* Work status, *CF* Cognitive function, *QSI* Quality of social interaction, *SexF* sexual function, *PS* Patient satisfaction, *DSE* Dialysis staff encouragement

Explanation: KDQOL^TM^ score: The quality of life of patients in the experimental group after 12 weeks was significantly higher than that of the control group before and after 12 weeks (p<0.01) in both KDTA and SF-36 total scores; Except for the four dimensions of SexF, SocF, BKD, and WS, which showed no significant differences, all other dimensions showed significant improvement compared to before (p<0.05); The control group showed no significant improvement in all other indicators except for the improvement in DSE and PS2 dimensions compared to before (P>0.05). Compared with the control group, after 12 weeks, the intervention group showed improvements in motor ability and KDQOLTM scores in PF, RP, Pain, GH, Energy, EWB, SPL, Sleeps, DSE, PS10 dimensions, as well as serum inflammatory indicators (Hs CRP, IL-1) and nutritional indicators (ALB and PA) compared to before (p<0.05). There was no statistically significant difference in the improvement of other indicators after intervention (see Tables 3 and 4).

### Kidney disease-related quality of life short form score

After 12 weeks, the patients' quality of life, as measured by KDQOL^TM^, was significantly higher in terms of KDTA and SF-36 total scores compared to before the intervention and in the control group (61.30 ± 5.38 vs 42.98 ± 5.73; 57.50 ± 3.55 vs 50.70 ± 3.62) (*p* < 0.01). Except for the SexF, SF, BKD and WS dimensions, the scores in other dimensions had improved significantly compared to before the intervention (*p* < 0.05). The improvement was particularly significant in the CF, SLE, RP, Pain and EWB dimensions (*p* < 0.01). In the control group, the improvement in most indicators was not significant, except for the DSE and PS dimensions (*p* > 0.05). When comparing the intervention with the control group, after 12 weeks, the intervention group showed improvements in physical abilities, KDQOL^TM^ scores in the PF, RP, CH, Energy, SPL, SL, DSE, Pain, CF, EWB and PS dimensions, as well as serum inflammation indicators (hs-CRP, IL-1) and nutrition indicators (ALB and PA), and the improvements were significant compared to before the intervention (*p* < 0.05). Other indicators did not show significant improvements (see Table 4).

**Table 4:** Comparison of nutritional status and inflammatory indicators between two groups of patients

Group	n	hs-CRP(mg/L)		IL-6(pg/ml)		TNF-α(pg/ml)		ALB(g/l)		PA(mg/L)	
		Baseline	End of study	Baseline	End of study	Baseline	End of study	Baseline	End of study	Baseline	End of study
Te-Group	25	8.93±0.99	3.02±0.34^△^	30.29±2.96	17.65±3.22^△^	56.47±13.23	55.14±15.36	30.62±1.65	35.60±1.68^△^	0.32±0.05	0.44±0.07^△^
Con-Group	24	8.79±0.98	8.98±1.09	30.21±4.24	29.65±3.08	56.71±16.28	57.63±11.21	30.16±1.50	31.28±2.03	0.32±0.06	0.34±0.07
t		0.509	-25.984	0.073	-13.325	-0.056	-0.644	1.015	8.118	-0.074	4.667
P		0.613	<0.001	0.942	<0.001	0.955	0.522	0.315	<0.001	0.941	<0.001

Data are mean ± SD. Within-group comparison with the baseline ^△^*P*<0.05; *ALB* albumin, *PA* Prealbumin, *IL-6* Interleukin-6, *hs-CRP* High-sensitivity C-reactive protein, *TNF* Tumor necrosis factor

Explanation: From the above table, it can be seen that after inter group comparison, there was no statistically significant difference in various indicators between the two groups before intervention, indicating comparability between the groups. The differences in hs CRP, IL-6, ALB, and PA before and after intervention in the intervention group were statistically significant, while the differences in all indicators before and after intervention in the control group were not statistically significant. After intervention, the differences in hs CRP, IL-6, ALB, and PA between the two groups were statistically significant.

## Discussion

Mindfulness meditation originates from Eastern Zen Buddhist philosophy. Its core principles are ‘conscious awareness’, ‘being present’ and ‘non-judgment’. Through various practices such as body scanning, mindful breathing and mindfulness meditation, it helps to enhance individual focus and improve emotional regulation, thereby helping to maintain emotional stability [40]. Clinical studies have found that mindfulness meditation not only helps alleviate depression and anxiety but also improves symptoms such as sleep disorders and pain in patients with chronic diseases. It also reduces fatigue and improves activity and health levels [41]. Progressive muscle relaxation training is a relaxation therapy that can quickly induce the body into a state of relaxation. It reduces skeletal muscle tension, increases blood flow to the skeletal muscles, decreases lactate levels, promotes metabolic synthesis and stimulates the secretion of related hormones, such as insulin and sex hormones. It also facilitates the recovery of organ and system functions. Progressive muscle relaxation training effectively compensates for the initial difficulty of those engaging in meditation to quickly enter a meditative state and serves as an auxiliary method before mindfulness meditation intervention [29].

The present study showed that after 12 weeks, in the combined intervention group, the 6-minute walking speed and grip strength increased compared to before in both the intervention and control groups, while the time taken to complete 10 sit-to-stand repetitions decreased (*p* < 0.05). The improvements in various indicators were not significant in the control group. However, there were no significant differences in SMI and SMM compared to the control group (*p* > 0.05). This suggests that short-term training during the dialysis interval effectively improved patients' physical function, enhanced exercise endurance and delayed the progression of sarcopenia. However, its effects on promoting muscle growth and increasing muscle mass were not significant. This may have been because both muscle relaxation training and mindfulness training release mental stress by relaxing the body’s muscles. Through breath training, as well as progressive muscle relaxation and other training methods to improve blood circulation, the body's resistance to disease can be improved and patients can be supported to better manage stress and relieve fatigue. Unlike resistance training, resistance exercise plays an important role in increasing muscle mass and muscle strength in the elderly. This may also have been due to the relatively short duration of the experiment and the small sample size; therefore, the results require further confirmation by clinical studies with larger sample sizes and a longer duration.

Research has shown that chronic inflammation is considered a driving factor in sarcopenia [42]. Inflammatory factors are associated with the activation of skeletal muscle pathway degradation, including the ubiquitin–proteasome system, the autophagy–lysosome system and apoptosis, leading to an imbalance between protein synthesis and breakdown, resulting in reduced muscle strength and mass [43]. Patients with uraemia often experience a state of low-grade inflammation [44]. Elevated levels of IL-6 and CRP significantly increase the risk of muscle loss in terms of muscle mass and strength, leading to imbalances in muscle tissue synthesis metabolism, increased protein breakdown and malnutrition [45]. Numerous clinical studies have confirmed that mindfulness meditation can alleviate inflammatory responses [46, 47]. In this study, it was observed that after 12 weeks, the combined intervention group showed a significant decrease in inflammatory markers CRP and IL-6 compared to before the intervention and the control group, with statistically significant differences. This suggests that the combined intervention had a positive effect on improving inflammation markers in haemodialysis patients. However, there were no significant differences in TNF-α inflammatory cytokine concentrations between the groups. Currently, there is conflicting research regarding the correlation between TNF-α and sarcopenia [48], and further research is needed to clarify this relationship. Another study found that the occurrence of haemodialysis-related sarcopenia was independently correlated with mood disorders. The higher the mood disorder score in patients receiving MHD, the higher the risk of developing sarcopenia (OR = −1.69, 95% CI: 1.14–2.51) [7]. Our previous animal trials found that restricted experiments (a modelling method used for depression animal models) on rats with uraemia could successfully create a sarcopenia animal model in the short term, indicating that mood disorders played a positive role in the development of sarcopenia [49]. Studies have proven that mindfulness meditation can also improve mood disorders [50, 51]. In this study, a 12-week intervention combining mindfulness meditation and progressive muscle relaxation training showed improvements in multiple dimensions of KDQOL^TM^, especially in cognitive function, emotional status and the impact of emotional problems on work and life (*p* < 0.01). The combined intervention effectively improved mood disorders and cognitive function and relieved emotional disorders, significantly enhancing the mental health of patients with sarcopenia. Additionally, there were significant effects in terms of reducing pain, improving sleep quality and enhancing physical work and life limitations, thereby considerably improving patients' quality of life. In terms of improvement in nutrition indicators (ALB, PA), this may have been related to the overall improvement in mood, increased appetite, reduced stress levels and decreased inflammation.

The participants of this study were patients with sarcopenia receiving haemodialysis (which involves aspects such as renal medicine and dialysis medicine) and who engaged in meditation, which required professional meditation experts. Accordingly, the intervention for these patients requires the joint cooperation and guidance of multidisciplinary and multi-field experts to ensure the safe and effective progress of the intervention procedure and ensure its clinical efficacy. Through the above results, it can be concluded that mindfulness meditation plus progressive muscle relaxation training can effectively improve the motor function ability and quality of life of patients. This method is simple and convenient; no intervention-related complications occurred during the study period, making it relatively safe. Moreover, it requires no additional costs and attempts can thus be made to popularise it in future clinical practice.

This study has several limitations. Mindfulness meditation has only gained widespread recognition and acceptance among scholars in recent years, and there is limited research data and findings available for reference. The standardisation of meditation procedures still needs improvement. Additionally, there were limitations in terms of sample size, limited indicators and control over influencing factors, which need further improvement in future experiments. No intervention-related complications occurred during the study period, indicating that the combined training method is safe and feasible.

## Conclusion

This study found that the combination of mindfulness meditation and progressive muscle relaxation training effectively improved the clinical efficacy and quality of life in patients with muscle-wasting syndrome. The mechanism of action may be related to the reduction of systemic inflammation and improvement in the patient's mood and nutritional status. Therefore, this combined intervention can be considered a simple, economical, safe and effective treatment method for patients with muscle-wasting sarcopenia undergoing haemodialysis. However, future clinical studies with larger sample sizes and longer duration may be needed to verify the efficacy and long-term effects of this study. Additionally, further studies on the association between TNF-α and sarcopenia are also needed to elucidate.

### Supplementary Information

Supplementary Material 1.

## Data Availability

The datasets used and/or analysed during the current study available from the corresponding author on reasonable request.

## References

[1] Cruz-Jentoft AJ, Baeyens JP, Bauer JM (2010). Sarcopenia: European consensus on definition and diagnosis: Report of the European Working Group on Sarcopenia in Older People. Age Ageing.

[2] GBD Chronic Kidney Disease Collaboration (2020). Global, regional, and national burden of chronic kidney disease, 1990–2017: a systematic analysis for the Global Burden of Disease Study 2017. Lancet.

[3] Ding J, Liu HJ, Huang X, Lian N (2021). Clinical manifestations of sarcopenia onset in maintenance hemodialysis patients and its relationship with TNF- α gene polymorphism. Chin J Integr Tradit Western Nephrol.

[4] Zhang Q, Qin HF, Jian GH, Cheng DS, Wang Z, Li JH, Wang W, Zhou TT, Wang NS (2020). Clinical characteristics and risk factors of sarcopenia in elderly hemodialysis patients. Chin J Geriatr.

[5] Ren H, Gong D, Jia F, Xu B, Liu Z (2016). Sarcopenia in patients undergoing maintenance hemodialysis: incidence rate, risk factors and its effect on survival risk. Ren Fail.

[6] Can B, Kara O, Kizilarslanoglu MC (2017). Serum markers of inflammation and oxidative stress in sarcopenia. Aging Clin Exp Res.

[7] Kurita N, Wakita T, Fujimoto S (2021). Hopelessness and depression predict sarcopenia in advanced CKD and dialysis: a multicenter cohort study. J Nutr Health Aging.

[8] Sieber CC (2019). Malnutrition and sarcopenia. Aging Clin Exp Res.

[9] Zalai D, Szeifert L, Novak M (2012). Psychological distress and depression in patients with chronic kidney disease. Semin Dial.

[10] Cukor D, Coplan J, Brown C (2007). Depression and anxiety in urban hemodialysis patients. Clin J Am Soc Nephrol.

[11] Hackett ML, Jardine MJ (2017). We need to talk about depression and dialysis: but what questions should we ask, and does anyone know the answers?. Clin J Am Soc Nephrol.

[12] Olagunju AT, Campbell EA, Adeyemi JD (2015). Interplay of anxiety and depression with quality of life in endstage renal disease. Psychosomatics.

[13] Kovacs AZ, Molnar MZ, Szeifert L (2011). Sleep disorders, depressive symptoms and health-related quality of life–a cross-sectional comparison between kidney transplant recipients and waitlisted patients on maintenance dialysis. Nephrol Dial Transplant.

[14] Peng T, Hu Z, Guo L, Xia Q, Li D, Yang X (2013). Relationship between psychiatric disorders and quality of life in nondialysis patients with chronic kidney disease. Am J Med Sci.

[15] Loosman WL, Rottier MA, Honig A, Siegert CE (2015). Association of depressive and anxiety symptoms with adverse events in Dutch chronic kidney disease patients: a prospective cohort study. BMC Nephrol.

[16] García-Llana H, Remor E, Del Peso G, Selgas R (2014). The role of depression, anxiety, stress and adherence to treatment in dialysis patients’ health-related quality of life: a systematic review of the literature. Nefrologia.

[17] Pompili M, Venturini P, Montebovi F (2013). Suicide risk in dialysis: review of current literature. Int J Psychiatry Med..

[18] Kabat-Zinn J (2003). Mindfulness-Based Interventions in Context: Past, Present, and Future. Clin Psychol Sci Pract.

[19] Eisendrath SJ, Gillung E, Delucchi KL (2016). A randomized controlled trial of mindfulness-based cognitive therapy for treatment-resistant depression. Psychother Psychosom.

[20] Zgierska A, Rabago D, Chawla N, Kushner K, Koehler R, Marlatt A (2009). Mindfulness meditation for substance use disorders: a systematic review. Subst Abus.

[21] Carlson LE (2012). Mindfulness-based interventions for physical conditions: a narrative review evaluating levels of evidence. ISRN Psychiatry.

[22] Heidari Gorji MA, Davanloo AA, Heidarigorji AM (2014). The efficacy of relaxation training on stress, anxiety, and pain perception in hemodialysis patients. Indian J Nephrol.

[23] Wang BQ (2023). The effect of short-term mindfulness meditation training on anxiety levels and coping styles in patients with generalized anxiety disorder. Psychol Month.

[24] Hernandez R, Burrows B, Browning M, et al. Mindfulness-based virtual reality intervention in hemodialysis patients: a pilot study on end-user perceptions and safety. Kidney360. 2021;2(3):435-444. 10.34067/kid.0005522020. 10.34067/KID.0005522020 PMC8786010 35369024

[25] Alhawatmeh H, Alshammari S, Rababah JA (2022). Effects of mindfulness meditation on trait mindfulness, perceived stress, emotion regulation, and quality of life in hemodialysis patients: a randomized controlled trial. Int J Nurs Sci.

[26] Rigas C, Park H, Nassim M (2022). Long-term effects of a brief mindfulness intervention versus a health enhancement program for treating depression and anxiety in patients undergoing hemodialysis: a randomized controlled trial. Can J Kidney Health Dis.

[27] Park J, Lyles RH, Bauer-Wu S (2014). Mindfulness meditation lowers muscle sympathetic nerve activity and blood pressure in African-American males with chronic kidney disease. Am J Physiol Regul Integr Comp Physiol.

[28] Mustata S, Cooper L, Langrick N, Simon N, Jassal SV, Oreopoulos DG (2005). The effect of a Tai Chi exercise program on quality of life in patients on peritoneal dialysis: a pilot study. Perit Dial Int.

[29] Talo B, Turan GB (2023). Effects of progressive muscle relaxation exercises on patients with epilepsy on level of depression, quality of sleep, and quality of life: A randomized controlled trial. Seizure.

[30] Yang ML, Wan YP, Chen S, Tan JX, Tang GD, Li YL, Wang S, Hu XP (2020). A review of progressive muscle relaxation training in cancer patients. Chin J Nurs Educ.

[31] Li XX, Zhang ZS, Bao X, Kong LX, Zhang L, Feng ZL, Wang ZH, Zhou HF, Liang WP, Gao JZ (2022). Progressive muscle relaxation trainings use for prevention and treatment of perinatal nausea and vomiting in neoadjuvant chemotherapy for breast cancer. Hebei Med J.

[32] Shi PZ (2020). Study on the effects of progressive muscle relaxation training on mood, sleep and quality of life in patients with hepatitis B cirrhosis. Inner Mongolia Med Univ.

[33] Zeng ZQ, Li QH, Lin MY (2018). Effect of progressive muscle relaxation training on fibromuscular pain syndrome and its improvement effect on the quality of life of patients. J Math Med.

[34] Liu WX. Effect of exercise rehabilitation on physical function of maintenance hemodialysis patients with sarcopenia. Chinese Journal of Nephrology, Dialysis & Transplantation. 2021;30(2):185-8. 10.3969/j.issn.1006-298X.2021.02.018.

[35] Chen LK, Liu LK, Woo J (2014). Sarcopenia in Asia: consensus report of the Asian Working Group for Sarcopenia. J Am Med Dir Assoc.

[36] Smith JC. Advances in ABC Relaxation: Applications and Inventories. Berlin: Springer Publishing Co; 2001. [Google Scholar]

[37] Wielgosz J, Goldberg SB, Kral TRA, Dunne JD, Davidson RJ (2019). Mindfulness meditation and psychopathology. Annu Rev Clin Psychol.

[38] Professional Committee of Tumor Nutrition, Chinese Anti-Cancer Association. Guidelines for Clinical Diagnosis and Treatment of Tumor-associated Sarcopenia. J Tumor Metab Nutr. 2022;9(01):24-34.

[39] Zou Y, Wen Y, Liu C, Qin X, Li GS (2018). A survey for depression and life quality in maintenance haemodialysis patients. Pract J Clin Med.

[40] Yu M, Yan YW, Lin RM, Cen RQ. Review of Neural Mechanism and Application Research on Mindfulness-Based Stress Reduction. J Fujian Normal Univ (Philos Soc Sci Edit), (in chinese). 2012;(6):209-216

[41] Bohlmeijer E, Prenger R, Taal E, Cuijpers P (2010). The effects of mindfulness-based stress reduction therapy on mental health of adults with a chronic medical disease: a meta-analysis. J Psychosom Res.

[42] Cruz-Jentoft AJ, Bahat G, Bauer J (2019). Sarcopenia: revised European consensus on definition and diagnosis. Age Ageing.

[43] von Haehling S, Ebner N, Dos Santos MR, Springer J, Anker SD (2017). Muscle wasting and cachexia in heart failure: mechanisms and therapies. Nat Rev Cardiol.

[44] Fuqiang L, Nephrology DO. Effect of hemodialysis and peritoneal dialysis on microinflammation of uremia patients in chronic renal failure and its relationship with cardiovascular disease. J Clin Intern Med. 2019.

[45] Cesari M, Kritchevsky SB, Baumgartner RN (2005). Sarcopenia, obesity, and inflammation–results from the Trial of Angiotensin Converting Enzyme Inhibition and Novel Cardiovascular Risk Factors study. Am J Clin Nutr.

[46] Malarkey WB, Jarjoura D, Klatt M (2013). Workplace based mindfulness practice and inflammation: a randomized trial. Brain Behav Immun.

[47] Lengacher CA, Kip KE, Barta M (2012). A pilot study evaluating the effect of mindfulness-based stress reduction on psychological status, physical status, salivary cortisol, and interleukin-6 among advanced-stage cancer patients and their caregivers. J Holist Nurs.

[48] Huang AL, Bu ZH, Xue MT, Li QY, Du SZ, Xu GH (2021). Peripheral inflammatory markers in the patients with sarcopenia: a meta-analysis. Chin J Osteoporosis.

[49] Wu YY, Yu RZ, Sun JH, Yan YH, Zhang HJ, Liu J, Qiu XH (2022). Modeling method and evaluation of myosthenia model in rats with uremia. Modern Pract Med.

[50] Brooks H, Oughli HA, Kamel L (2021). Enhancing Cognition in Older Persons with Depression or Anxiety with a Combination of Mindfulness-Based Stress Reduction (MBSR) and Transcranial Direct Current Stimulation (tDCS): Results of a Pilot Randomized Clinical Trial. Mindfulness (N Y).

[51] Kim DY, Hong SH, Jang SH (2022). Systematic Review for the Medical Applications of Meditation in Randomized Controlled Trials. Int J Environ Res Public Health..

